# A case report of persistent cerebellar dysfunction following acute lithium toxicity

**DOI:** 10.1186/s12883-026-04788-7

**Published:** 2026-03-09

**Authors:** Jiabei Nie, Qing Gao, Yiming Li, Xiaomeng Xu, Yuyan Tan

**Affiliations:** 1 Department of Neurology and Institute of Neurology, Ruijin Hospital, Shanghai Jiao Tong University School of Medicine, Shanghai, 200025 China; 2 Department of Neurology, Wuxi Branch of Ruijin Hospital, Shanghai Jiao Tong University School of Medicine, Wuxi, 214000 China; 3 Department of Neurology, Baotou Central Hospital, Baotou, 014040 China

**Keywords:** Lithium toxicity, Ataxia, Cerebellar atrophy, Neurotoxicity, NMS-like syndrome

## Abstract

**Background:**

Acute lithium toxicity can cause cerebellar impairment, but long-term follow-up data are limited. We describe a case of acute lithium intoxication followed by progressive cerebellar atrophy and permanent cerebellar dysfunction.

**Case presentation:**

Five years ago, a previously healthy 38-year-old man developed a clinical syndrome consistent with neuroleptic malignant syndrome (NMS)-like syndrome in the setting of acute lithium and quetiapine toxicity. On admission, the serum lithium concentration was 4.07 mmol/L and the cerebrospinal fluid lithium concentration was 2.45 mmol/L. Continuous renal replacement therapy (CRRT) was initiated, and cerebrospinal fluid lavage was also performed as documented in the outside hospital records. Serum lithium levels subsequently decreased to below 0.2 mmol/L. After treatment, consciousness improved. However, he developed a persistent cerebellar syndrome, characterized by ataxia, gait instability, and dysarthria. Brain magnetic resonance imaging one year later revealed cerebellar atrophy. Over five years of follow-up, severe ataxia persisted, and serial MRI showed progressive isolated cerebellar atrophy without supratentorial structural abnormalities.

**Conclusions:**

This case suggests that severe acute lithium toxicity may be associated with progressive cerebellar degeneration, even after normalization of serum lithium levels. Clinicians should consider long-term follow-up for neurological sequelae in individuals suffering from acute lithium intoxication.

**Supplementary Information:**

The online version contains supplementary material available at 10.1186/s12883-026-04788-7.

## Background

Lithium salts are a cornerstone of treatment for bipolar disorder, but their therapeutic window is narrow and carries a high risk of toxicity [1, 2]. Both an acute overdose and chronic poisoning can cause lithium intoxication for a variety of reasons. Drug interactions, prescription or dispensing errors, coexisting conditions that impair renal function, or chronic conditions that result in fluid loss, such as dehydration and lithium-induced nephrogenic diabetic insipidus, are some of these causes [3]. Although lithium can be harmful to many different systems, the central nervous system (CNS) is usually where its most notable effects are seen. Lithium poisoning can cause a variety of acute and long-term neurological symptoms, the most common of which are tremors. Encephalopathy, which manifests as altered mental status, slurred speech, ataxia, and nystagmus, can happen at any point throughout treatment [4]. Neuromuscular effects include proximal muscle weakness, muscle breakdown, myasthenia gravis-like symptoms, and axonal neuropathy [5–7]. These neurological symptoms are usually reversible and improve with reduction or discontinuation of the drug and persistent neurological deficits are rare [4]. Persistent cerebellar toxicity is considered an uncommon consequence of lithium treatment [5, 6]. In some cases, cerebellar symptoms such as ataxia, tremor, and dysarthria may persist even after lithium levels return to normal [4]. The combined use of lithium with antipsychotics may increase the risk of neurotoxicity, leading to more severe neurological damage [5]. Some studies have shown that in the presence of complications such as renal insufficiency or fever, patients may develop irreversible lithium-effect neurotoxicity syndrome. Adityanjee proposed the term syndrome of irreversible lithium-effectuated neurotoxicity(SILENT) [8] to describe these manifestations characterized by persistent cerebellar symptoms and radiographic cerebellar atrophy [9, 10].

Here we present a case of persistent cerebellar dysfunction following acute lithium toxicity, and discusses its clinical features in conjunction with relevant literature, aiming to improve the diagnosis and recognition of lithium toxicity-induced cerebellar ataxia.

## Case presentation

The patient was a previously healthy 38-year-old man with no significant medical history. Notably, he had no prior history of lithium use and no psychiatric indication for lithium therapy. On November 4, 2020, he was found by his family unconscious and unresponsive. They found that he had taken a large quantity of medication while drunk at home: about 240 tablets of 100 mg quetiapine extended-release and 100 tablets of 0.25 g lithium carbonate, for reasons that remain unclear.

**Fig. 1:**
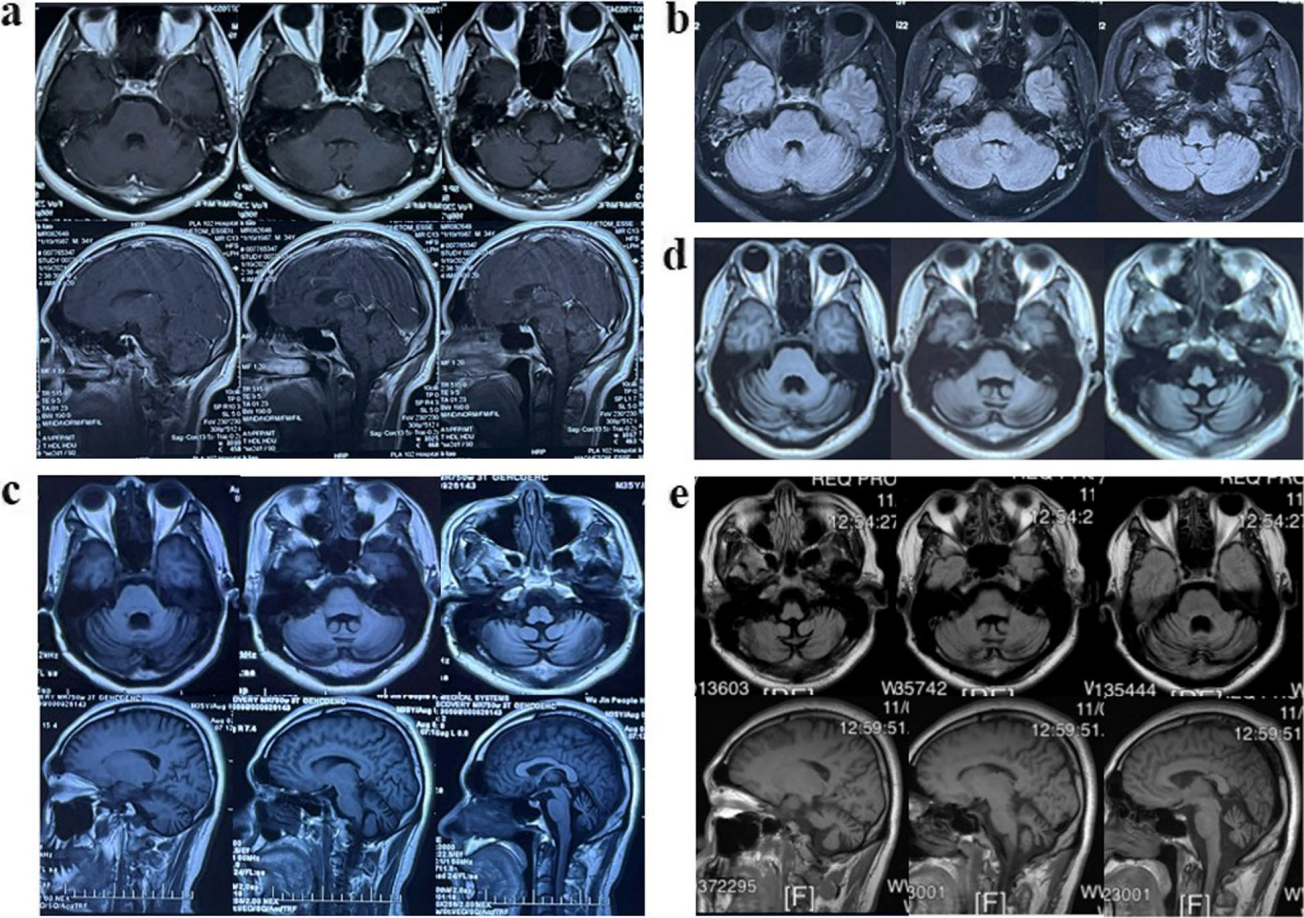
Panels **a-e** show head MRI scans from January 2021 (**a**), December 2021 (**b**), August 2022 (**c**), December 2023 (**d**), and November 2025 (**e**), respectively. These images show the continued progression of cerebellar atrophy without damage to other brain structures

He was immediately taken to a local hospital for emergency gastric lavage, during which a large amount of tablet fragments was retrieved. Antibiotics and fluid resuscitation were subsequently administered. The patient was able to move on his own after regaining consciousness, but he was unable to recall the time he had been unconscious. On hospital day 4, he developed altered mental status, generalized tonic–clonic seizures, intermittent fever, and increased muscle tone with generalized rigidity on examination. After diazepam was administered for sedation, the symptoms subsided. He then experienced recurring convulsions and bronchospasm, a high fever with a peak temperature of 42 °C, and transient oxygen desaturation to 50% during convulsive episodes, which normalized after seizure control. Mechanical ventilation was initiated for airway protection in the setting of impaired consciousness and seizures, rather than primary respiratory failure. According to pertinent tests (November 9, 2020), the patient's cerebrospinal fluid(CSF) lithium concentration was 2.45 mmol/L (normal range 0.3–0.5 mmol/L) and serum lithium concentration was 4.07 mmol/L (normal range 0.6–1.2 mmol/L). These samples were obtained before extracorporeal therapy. The patient presented with high fever, dehydration, bacterial infection, acute kidney injury(AKI), acute liver injury, and rhabdomyolysis, according to the clinical signs and laboratory results (Table 1). Creatine kinase was markedly elevated. Given an overdose of dopamine antagonist exposure, hyperthermia, altered consciousness, muscle rigidity, and elevated creatine kinase, the presentation was considered compatible with an NMS-like hyperthermic rhabdomyolysis syndrome in the setting of severe acute lithium and quetiapine intoxication. However, sustained autonomic instability was not clearly documented in the available medical records. Head CT scans, performed on November 6 and November 11, 2020(Fig. S1) were unremarkable. MRI was not feasible during the acute phase due to unstable clinical status and seizure. The first post-stabilization brain MRI, obtained on January 19, 2021 (Fig. 1a), was normal, demonstrating no basal ganglia involvement, no cortical or cerebellar signal abnormalities across all sequences (DWI, T2 FLAIR, T1). According to medical records, there was no documented cardiac arrest or sustained hypotension, and aside from transient desaturation during seizures, no evidence of prolonged global cerebral hypoperfusion. A chest CT scan showed unequal pleural thickening and bilateral lower lobular inflammation. The referring hospital’s records indicated that the patient underwent continuous renal replacement therapy(CRRT) on November 10, a CSF lavage on November 11, and lumbar cistern drainage on November 13, 2020.

**Table 1:** Initial laboratory findings at hospital admission (Pre-CRRT)

Laboratory Test	Patient Value	Normal Levels
Lithium Level Test		
Serum Lithium(mmol/L)	4.07	0.6—1.2
CSF Lithium(mmol/L)	2.45	0.3—0.5
Complete Blood Count		
Hemoglobin(g/L)	177	130—175(Male)
Hematocrit	0.52	0.40—0.50(Male)
White Blood Cell count	21.6 × 10^9^	4.0—11.0 × 10^9^
Neutrophil percentage(%)	79.9	40—75
Blood Biochemistry		
Myoglobin(ng/mL)	> 3904.0	< 70
CTnI(ng/mL)	0.838	< 0.04
Creatine Kinase(U/L)	3700	50—200(Male)
CK-MB(ng/mL)	36.9	< 5 ng/mL
LDH(U/L)	500	120—240
Chloride (mmol/L)	108.8	98—107
SCr(μmol/L)	737.6	62—115(Male)
BUN(mmol/L)	22.8	2.5—7.1
Na^+^ (mmol/L)	151.7	135—145
ALT(U/L)	76	< 40
AST(U/L)	160	< 40
Ca^+^(mmol/L)	2.72	2.10—2.55
CRP(mg/L)	26.4	< 5
Coagulation Tests		
Fibrinogen (g/L)	6.40	2.0—4.0
PT(s)	14.2	11—14
D-dimer(mg/L FEU)	0.87	< 0.5
PCT(ng/mL)	53.2	< 0.05

*CSF* cerebrospinal fluid, *CTnI* cardiac troponin I, *CK-MB* creatine kinase-MB, *LDH* lactate dehydrogenase, *SCr* serum creatinine, *BUN* blood urea nitrogen, *ALT* alanine aminotransferase, *AST* aspartate aminotransferase, *CRP* C-Reactive Protein, *PT* prothrombin time, *PCT* procalcitonin

After more than 20 days of treatment, the patient regained consciousness. On Nov 18, 2020, serum and CSF lithium levels gradually decreased to less than 0.2 mmol/L, and liver and kidney function indicators returned to normal. However, symptoms of dysarthria, dizziness, vertigo and diplopia during movement, dysphagia, difficulty sitting up, and inability to walk remained. Follow-up examinations in 2021 showed that the serum lithium concentration was normal. During subsequent treatment, the patient received regular rehabilitation therapy and took idebenone 1 tablet three times a day and buspirone 2 tablets three times a day. Pharmacologic therapy included idebenone for potential neuroprotective and mitochondrial support, and buspirone to improve cerebellar ataxia and alleviate residual anxiety. Rehabilitation therapy focused on balance, gait, and coordination training to support cerebellar function and improve independence in daily activities. His dysarthria, limb weakness, and unsteady gait gradually improved, and he was able to stand and walk with assistance. However, his rehabilitation progress entered a plateau phase since 2024, and by November 2025, the remaining symptoms included slurred speech, double vision, dizziness, and unsteady gait (Video. 1). Physical examination revealed dysarthria, gaze-evoked nystagmus in horizontal and vertical directions, impaired smooth pursuit, saccadic dysmetria, and diplopia (Video. 2). Functionally, the patient was able to stand independently, walk with assistance, and perform daily activities with partial independence. Structured ataxia assessment (SARA) at this time yielded a total score of 21/40, indicating moderate-to-severe ataxia. In November 2025, whole-exome sequencing and dynamic mutation detection of spinal cerebellar ataxia(SCA) showed no abnormalities. Routine blood tests, liver and kidney function tests, electrolytes levels, and creatine kinase levels were normal. Serial head MRIs from January and December 2021 (Fig. 1a and b), August 2022 (Fig. 1c), December 2023 (Fig. 1d), and November 2025 (Fig. 1e) demonstrated progressive, isolated cerebellar atrophy without supratentorial involvement, supporting selective cerebellar degeneration likely related to lithium neurotoxicity. The case timeline of key events and interventions is presented in Supplementary Table 1.

## Discussion and conclusions

Lithium remains a cornerstone therapy for bipolar disorder but has a narrow therapeutic index. Lithium toxicity may present as acute, acute-on-chronic, or chronic poisoning [11, 12]. The present case represents acute lithium toxicity in a lithium-naive individual. The risk of neurotoxicity is often higher in cases of chronic poisoning than in acute poisoning. This difference is thought to reflect intracellular lithium accumulation within the central nervous system(CNS), influenced by the plasma concentration–time profile. Acute toxicity may cause tremor, ataxia, dysarthria, seizures, encephalopathy, or coma. Symptom severity often correlates with serum lithium concentrations [13, 14].

In this case, a 38-year-old man developed severe acute lithium toxicity following accidental overdose. The patient's symptoms initially improved after receiving quick treatment that included gastric lavage, intravenous fluids, antibiotic therapy and CRRT. Long after the serum lithium levels returned to normal, the patient continued to exhibit progressive cerebellar atrophy and persistent cerebellar symptoms, including ataxia, dysarthria, and severe gait instability. These deficits were temporally associated with the acute lithium overdose—a phenomenon described in the literature as Syndrome of Irreversible Lithium-Effectuated Neurotoxicity(SILENT). Nevertheless, other factors may have contributed. The patient experienced hyperthermia (42 °C), transient seizure-related oxygen desaturation and mechanical ventilation. However, there was no documented sustained hypotension, cardiac arrest, or evidence of prolonged hypoxemia. Early CT scans were unremarkable, and the first MRI obtained after clinical stabilization showed no abnormalities. Serial imaging later revealed progressive, isolated cerebellar atrophy without supratentorial involvement. Despite this, the potential contributory effects of the systemic illness cannot be entirely excluded.

Lithium poisoning can have mild to severe early symptoms. Disorientation, lack of coordination, seizures, and encephalopathy are important signs of neurotoxicity. Previous studies have shown that those with neurotoxic effects and lithium toxicity do not fully recover [3, 15–17]. Persistent neurological deficits following lithium intoxication have been described as SILENT. Ninety examples of neurological impairments lasting longer than two months following lithium intoxication were found in a study of the literature by Adityanjee et al. [8]. Ataxia, dysarthria, and dysmetria were among the signs of persistent cerebellar dysfunction that were present in most of the patients in this cohort. Further investigations of patients with persistent cerebellar symptoms after lithium toxicity have revealed irreversible cerebellar damage, as evidenced by computed tomography [8], magnetic resonance imaging [18], and histological findings [19], including neuronal loss and gliosis in the cerebellar gray matter.

According to some research, cerebellar impairment brought on by lithium poisoning typically develops gradually in patients who use lithium for a long time [20, 21]. Typically, neurological symptoms from acute lithium toxicity are reversible once lithium levels return to therapeutic range. In the 1970 s, chronic cerebellar damage following acute lithium toxicity was first reported, with patients presenting with persistent ataxia, tremor, and dysarthria [22]. Acute poisoning from high lithium concentrations might cause long-term symptoms, such as limb and truncal ataxia, choreoathetosis, or parkinsonism, according to later case reports and case series [4, 21, 23]. During therapy, these long-lasting toxic effects may worsen, especially if there is a high fever, dehydration, or antipsychotic drug use, all of which may contribute to the toxicity's persistence [7, 13, 22, 24–26]. After the initial intoxication has subsided, neurological sequelae usually appear. The most common indication is cerebellar dysfunction, although damage is frequently seen at multiple locations within the nervous system [16, 27].

Acute lithium exposure may lead to prolonged neurological impairment even after serum levels normalize. Its neurotoxic mechanisms are incompletely understood. The distribution of lithium was complex. Organs such as the liver, kidneys, thyroid, and bones absorb lithium relatively quickly, while it takes longer for lithium to reach stable concentrations in the brain [28, 29]. Brain lithium concentrations may exceed serum levels due to intracellular accumulation [19]. The precise molecular mechanism of neurotoxicity is unknown; nevertheless, biopsy results have revealed significant demyelination in the afflicted peripheral nerves [30–32]. It is likely that demyelination at multiple sites within the CNS, particularly in the cerebellum, contributes to the persistence of neurological sequelae, including Purkinje cell loss [33], gliosis of the cerebellar cortex [19], and cerebellar atrophy. Experimental studies have shown that lithium interacts with the processes that govern calcium input into Purkinje cells, causing excitotoxic effects [34]. Nevertheless, the mechanisms underlying this selective effect remain unknown and require further investigation.

In this case, the patient had a single accidental high-dose lithium exposure, and lithium was rapidly eliminated by gastric lavage and CRRT. According to the referring hospital’s records, the patient also underwent CSF lavage on November 11 and lumbar cistern drainage on November 13. While detailed information on session duration, timing relative to peak lithium levels, and serial lithium measurements was not available. According to recommendations from the EXTRIP Workgroup, intermittent hemodialysis is generally preferred for severe lithium poisoning, with CRRT as an alternative when hemodialysis is not feasible. CSF lavage and lumbar cistern drainage in this case were aimed at facilitating reduction of lithium concentration in the CSF compartmen. These interventions are not standard treatments for lithium poisoning and are not routinely recommended in current guidelines; however, they were applied based on local clinical judgment in this critically ill patient. The evidence supporting CSF-targeted interventions for lithium clearance is limited, and their use in this case was primarily to address the severity of neurological involvement. The successful lithium clearance was confirmed by repeated serum and CSF lithium level tests. Despite effective lithium clearance, prolonged neurological impairment developed, suggesting that early toxic injury may initiate downstream pathological processes. Studies have shown that lithium may have neurotoxic effects on the cerebellum, particularly affecting Purkinje cells, leading to spongiform degeneration of the cerebellar white matter and subsequently cerebellar dysfunction [18]. High concentrations of lithium can alter neuronal function, causing cellular damage that may be irreversible [18, 19, 34].

Our case suggest that severe acute lithium toxicity may be associated with irreversible cerebellar dysfunction. In conclusion, this case highlights that severe acute lithium toxicity, even in a lithium-naive patient, may be followed by persistent cerebellar dysfunction and cerebellar atrophy. While causality cannot be definitively established and confounding factors must be acknowledged, the temporal relationship and selective imaging findings support lithium-associated cerebellar degeneration. Further studies are required to clarify the mechanisms linking acute lithium toxicity to chronic cerebellar degeneration and to explore therapeutic strategies beyond lithium removal.

## Supplementary Information

Supplementary Material 1: Video 1. Cerebellar ataxia caused by lithium intoxication. The ataxia is characterized by gait instability with a wide-based gait, impaired balance, a tendency to fall laterally or backward, and a drunken-like gait.

Supplementary Material 2: Video 2. Ocular motor abnormalities associated with cerebellar atrophy following lithium intoxication. Gaze-evoked nystagmus is observed during sustained eccentric gaze, with increasing amplitude as the gaze deviates further from the primary position.

Supplementary Material 3: Supplementary Table 1. Case Timeline of Key Events and Interventions.

Supplementary Material 4: Fig.S1 Head CT scans from the acute phase. Head CT scans performed on November 6, 2020(a) and November 11, 2020(b), were unremarkable.

## Data Availability

Data sharing is not applicable to this article as no datasets were generated or analysed during the current study.

## Abbreviations

AKI: Acute kidney injury
CNS: Central nervous system
CRRT: Continuous renal replacement therapy
CSF: Cerebrospinal fluid
NMS: Neuroleptic malignant syndrome
SCA: Spinal cerebellar ataxia
SILENT: Syndrome of Irreversible Lithium-Effectuated Neurotoxicity
