# Supplementary material for: A case report of persistent cerebellar dysfunction following acute lithium toxicity
Source: BMC Neurol. 2026 Mar 9;26:252. doi: 10.1186/s12883-026-04788-7 (PMC13085407; doi:10.1186/s12883-026-04788-7)
Supplement: Supplementary file 3 — Supplementary Material 3: Supplementary Table 1. Case Timeline of Key Events and Interventions. [file 12883_2026_4788_MOESM3_ESM.pdf]

Date	Event	Lab / Imaging	Intervention/ Notes
Nov 4, 2020	Lithium and quetiapine overdose<br>Emergency admission	—	Gastric lavage
Nov 6, 2020	—	Head CT	Head CT: unremarkable
Nov 9, 2020	Hyperthermia, AKI, liver injury, rhabdomyolysis,<br>Intermittent seizures<br>ICU admission	Serum Li 4.07 mmol/L<br>CSF Li 2.45 mmol/L	Mechanical ventilation
Nov 10, 2020	—	—	CRRT initiated
Nov 11, 2020	—	Head CT	Head CT: unremarkable<br>CSF lavage
Nov 13, 2020	—	—	Lumbar cistern drainage
Nov 18, 2020	—	Serum Li <0.2 mmol/L<br>CSF Li <0.2 mmol/L	CRRT discontinued
Nov 18, 2020	—		Extubation
Nov 26, 2020	Clinical stabilization<br>Cerebellar ataxia	—	Liver/Kidney function normalized
Jan, 2021<br>Dec, 2021	Progressive cerebellar ataxia	Brain MRI	Rehabilitation and Pharmacotherapy<br>Brain MRI: unremarkable
Aug, 2022		Brain MRI	Rehabilitation and Pharmacotherapy<br>Brain MRI: progressive, isolated cerebellar atrophy without supratentorial involvement
Dec, 2023		Brain MRI	
Mar, 2025		Brain MRI	
