# Supplementary figures and images for: A case report of persistent cerebellar dysfunction following acute lithium toxicity
Source: BMC Neurol. 2026 Mar 9;26:252. doi: 10.1186/s12883-026-04788-7 (PMC13085407; doi:10.1186/s12883-026-04788-7)

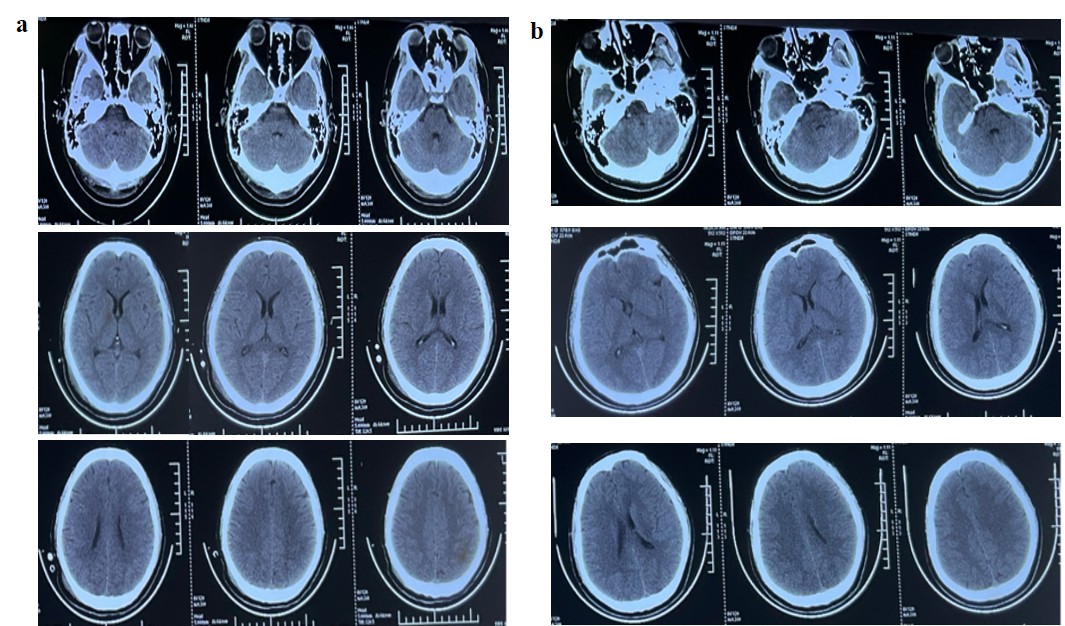

Supplement: Supplementary file 4 — Supplementary Material 4: Fig.S1 Head CT scans from the acute phase. Head CT scans performed on November 6, 2020(a) and November 11, 2020(b), were unremarkable. [file 12883_2026_4788_MOESM4_ESM.jpg]
